# A Profusion of Molecular Scissors for Pectins: Classification, Expression, and Functions of Plant Polygalacturonases

**DOI:** 10.3389/fpls.2018.01208

**Published:** 2018-08-14

**Authors:** Yang Yang, Youjian Yu, Ying Liang, Charles T. Anderson, Jiashu Cao

**Affiliations:** ^1^Laboratory of Cell and Molecular Biology, Institute of Vegetable Science, Zhejiang University, Hangzhou, China; ^2^Key Laboratory of Horticultural Plant Growth, Development and Quality Improvement, Ministry of Agriculture – Zhejiang Provincial Key Laboratory of Horticultural Plant Integrative Biology, Hangzhou, China; ^3^Department of Horticulture, College of Agriculture and Food Science, Zhejiang A & F University, Hangzhou, China; ^4^Department of Biology, The Pennsylvania State University, University Park, Pennsylvania, PA, United States; ^5^Center for Lignocellulose Structure and Formation, The Pennsylvania State University, University Park, Pennsylvania, PA, United States

**Keywords:** polygalacturonase, pectin modification, cell wall, classification, expression pattern, function

## Abstract

In plants, the construction, differentiation, maturation, and degradation of the cell wall are essential for development. Pectins, which are major constituents of primary cell walls in eudicots, function in multiple developmental processes through their synthesis, modification, and degradation. Several pectin modifying enzymes regulate pectin degradation via different modes of action. Polygalacturonases (PGs), which function in the last step of pectin degradation, are a crucial class of pectin-modifying enzymes. Based on differences in their hydrolyzing activities, PGs can be divided into three main types: exo-PGs, endo-PGs, and rhamno-PGs. Their functions were initially investigated based on the expression patterns of PG genes and measurements of total PG activity in organs. In most plant species, PGs are encoded by a large, multigene family. However, due to the lack of genome sequencing data in early studies, the number of identified PG genes was initially limited. Little was initially known about the evolution and expression patterns of PG family members in different species. Furthermore, the functions of PGs in cell dynamics and developmental processes, as well as the regulatory pathways that govern these functions, are far from fully understood. In this review, we focus on how recent studies have begun to fill in these blanks. On the basis of identified PG family members in multiple species, we review their structural characteristics, classification, and molecular evolution in terms of plant phylogenetics. We also highlight the diverse expression patterns and biological functions of PGs during various developmental processes, as well as their mechanisms of action in cell dynamic processes. How PG functions are potentially regulated by hormones, transcription factors, environmental factors, pH and Ca^2+^ is discussed, indicating directions for future research into PG function and regulation.

## Introduction

The cell walls of plants are unique extracellular structures, the construction, differentiation, maturation, and degradation of which lay the foundations for tissue differentiation, organ patterning and developmental transitions. Their structural variability in different plant species not only reflects the phylogenetic diversity of plants, but is also associated with the complexity of plant development and plant resistance to biotic and abiotic stresses (Somerville et al., 2004; Cosgrove, 2005; Braybrook and Joensson, 2016). In primary cell walls, 90% of the non-water mass is composed of cellulose, hemicelluloses, and pectins (Albersheim et al., 1996). Pectins, which are acidic polysaccharides that surround cellulose and hemicelluloses in a matrix, control wall porosity, wall hydration, and intercellular adhesion (Daher and Braybrook, 2015; Anderson, 2016). They can be classified into homogalacturonan (HG), rhamnogalacturonan-I, rhamnogalacturonan-II, and xylogalacturonan domains (Sénéchal et al., 2014). As major constituents of primary cell walls, the pollen intine, pollen tube walls, and the middle lamella (Aouali et al., 2001; Dardelle et al., 2010), pectins and their synthesis and degradation influence tissue elongation, pollen development, fruit ripening, and organ abscission (Willats et al., 2001; Sénéchal et al., 2014). The metabolism of pectins in the cell wall is regulated by different classes of pectin-modifying enzymes (Willats et al., 2001; Sénéchal et al., 2014). The polygalacturonases (PGs), which comprise one of these classes, catalyze the hydrolysis of pectins and are involved in numerous developmental processes. Based on differences in hydrolyzing activity, PGs can be divided into three main types: exo-PGs, endo-PGs, and rhamno-PGs. Therefore, illuminating the functions of PGs is of great importance in understanding the dynamics of cell walls during plant growth and strengthening breeding strategies for improving the productivity of crop varieties.

Early studies succeeded in cloning PG genes from species like maize (*Zea mays*) (Allen and Lonsdale, 1993), tobacco (*Nicotiana tabacum*) (Tebbutt et al., 1994), peach (*Prunus persica*) (Lester et al., 1996), and tomato (*Solanum lycopersicum*) (Kalaitzis et al., 1997). Total PG activity was measured in various organs of these species, indicating the functions of PGs in fruit ripening, pollen development, and organ abscission, in line with the expression patterns of these genes. Hadfield and Bennett (1998) reviewed this initial progress and proposed the first classification system for PGs (Hadfield et al., 1998). However, without genome data, little was known about the composition, classification, and evolution of the PG family. Although the sequences of some PG genes were reported, only a few of these were characterized in terms of their expression patterns and functions. At the turn of the 21st century, it remained unclear how pectin hydrolysis by PGs alters cell wall structure, modulates cell growth, and regulates organ growth.

In the last 18 years, the expression patterns and putative functions of hundreds of PG genes have been delineated. Some regulatory factors for PGs at both the transcriptional and post-translational levels have also been reported. Innovative research methods and a flood of genome sequencing data have both contributed to the systematic identification and phylogenetic analysis of PG families in numerous plant species. This review highlights the latest advances in the structural analysis and classification of PG families, the molecular evolution of PG genes in the context of plant evolution, and the expression, functions, and regulators of PGs during different developmental processes in plants. It also explores the basis for a better understanding of how cell wall dynamics influence cell and plant growth.

## Structural Characteristics and Classification of Polygalacturonases

### Structural Characteristics of Polygalacturonases

Polygalacturonases belong to glycoside hydrolase family 28 and contain at least one GH28 (Pfam00295) domain (Markovič and Janeček, 2001; Kim et al., 2006). This domain is replaced by a Pectate Lyase 3 domain (Pfam12708) in Arabidopsis (*Arabidopsis thaliana*) *QRT3* and its homologs in other species (Rhee et al., 2003; Yu et al., 2014). The encoded protein of Arabidopsis *QRT3* exhibits PG activity, as demonstrated by heterologous expression in *Saccharomyces cerevisiae* (Rhee et al., 2003). Most PG genes (91.2% of PG genes in Arabidopsis and 87.9% of PG genes in Chinese cabbage (*Brassica campestris*, syn. *Brassica rapa*) are predicted to encode a signal peptide upstream of the GH28 domain. Since signal peptides typically function in guiding proteins through secretory pathways that end with exocytosis (Babu et al., 2013), their presence implies that most PGs should be located in the apoplast. This hypothesis has been supported in recent studies of PG localization (Irshad et al., 2008; Xiao et al., 2014, 2017; Rui et al., 2017). In addition to the apoplast, one PG with a signal peptide has been localized in Golgi bodies and vesicles *in vitro*, presumably as part of its secretory journey (Nakashima et al., 2004).

In plant and fungal PGs, there are four commonly conserved, functional domain motifs known as SPNTDG (motif I), GDDC (motif II), CGPGHGISIGSLG (motif III), and RIK (motif IV), although domain III shows lower conservation and is missing in rhamno-PGs (Park et al., 2008). Typically, a PG gene encodes at least one of these domains (Torki et al., 2000). Within these four motifs, NTD, DD, GHG, and RIK amino acid segments are conserved active-site residues in plant, fungal, bacterial, and insect PGs, and have been demonstrated to be essential for fungal PG activity (Bussink et al., 1991; Markovič and Janeček, 2001). Within motifs I and II, the aspartic acid (D) residues in NTD and DD are components of the catalytic site (Rexovabenkova, 1990). In motif III, the histidine (H) residue acts in catalysis (Rao et al., 1996; Markovič and Janeček, 2001). Finally, the positively charged motif IV is thought to form interactions with the carboxylate groups of pectate substrates (Bussink et al., 1991).

**FIGURE 1:**
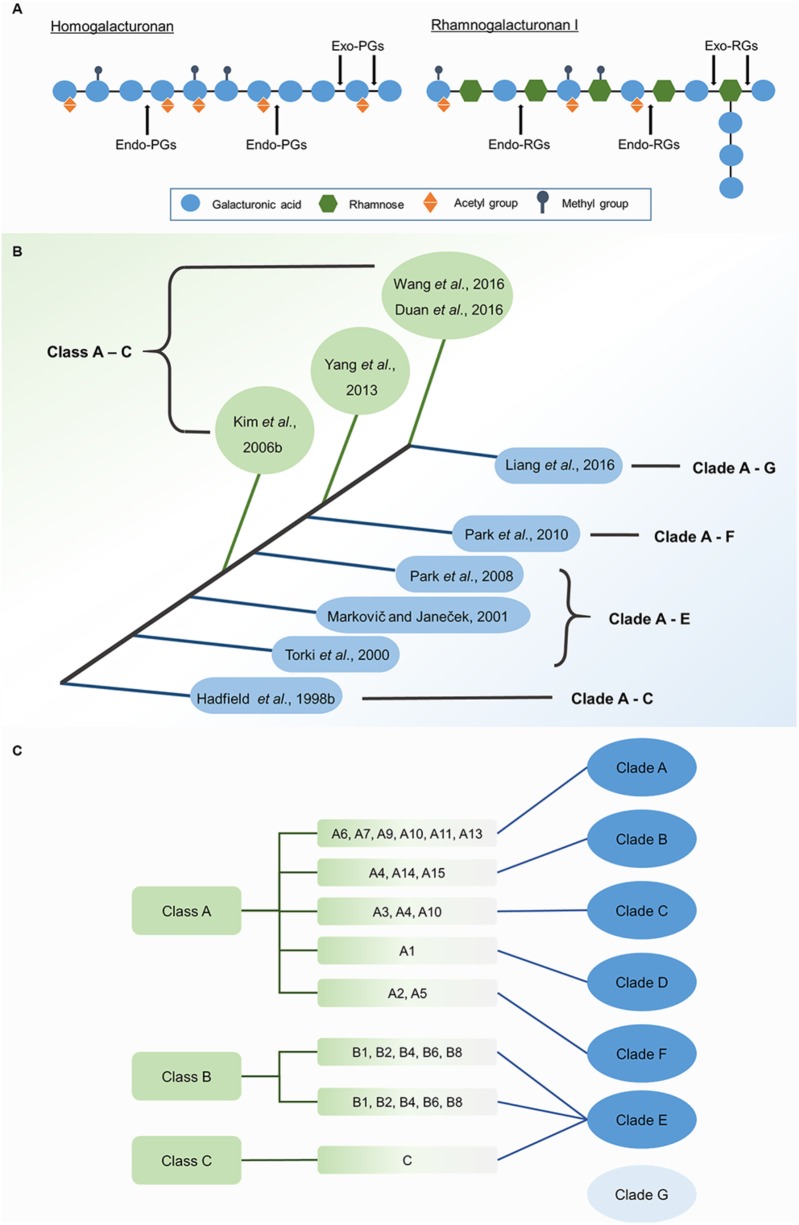
Modes of action of PGs of different types **(A)** and classification of PG genes **(B)**. **(A)** Different types of PGs differ in the selection of substrates and the action site of pectins main chain. **(B)** Based on the system first proposed by Kim, PG genes were all grouped into three classes (with green background). Based on the system first proposed by Hadfield, PG genes were divided into three to seven clades in different studies (with blue background). **(C)** A comparison of Kim’s system (with green background) and Liang’s system (with blue background) in the classification of Arabidopsis PG gene family.

Crystal structures of PGs can reveal the mechanisms by which they select substrates or act in catalysis. Although crystal structures of plant PGs are not yet published, we can infer their modes of action by inspecting the reported structures of fungal and bacterial enzymes due to the high conservation of functional domains between plant, fungal, and bacterial PGs. A typical PG displays 10 complete turns of β-structure, which is formed by four parallel sheets extending along the longitudinal axis. This structure can be used to distinguish PGs from polysaccharide lyases, which have three-sheet topology (Kluskens et al., 2005; Abbott and Boraston, 2007). Endo-PGs and exo-PGs show significant differences in crystal structures, indicating their different substrates and modes of action (**Figure 1A**). The active site of a typical endo-PG is a tunnel-like substrate-binding cleft lying between two loop regions. Hence, endo-PGs can potentially bind polysaccharides in either direction and produce oligosaccharides with varying degrees of polymerization (Jenkins et al., 1998). The adjacent loop regions help identify substrates and guide them to the active site (Pickersgill et al., 1998). For example, in *Achaetomium* sp. Xz8 endo-PG, the Asn94 residue of the T3 loop binds to substrates in the active site cleft by forming a hydrogen bond, which ensures correct positioning of substrates (Tu et al., 2015). In contrast, exo-PGs have a closed-pocket active site that only binds to the non-reducing ends of pectins due to inserted stretches of amino acids (Abbott and Boraston, 2007). Rhamno-PGs (RGs), which hydrolyze GalA-rhamnose bonds of rhamnogalacturonan-I, can be further divided into exo-RGs and endo-RGs (Mutter et al., 1998; Choi et al., 2004; Damak et al., 2015). However, tertiary structures of exo-RGs remain unreported. A predicted structure of an endo-RG has only been modeled in *Aspergillus aculeatus* (Choi et al., 2004). Compared with endo-PGs, endo-RGs are also predicted to have a tunnel-like active-site with two open ends. Differences in loop structure are likely to provide endo-RGs with more space in the active site to bind more complex substrates. The most significant difference between the structures of endo-PGs and endo-RGs is that endo-RGs have long tails of 19 and 45 residues at the N-terminus and C-terminus, respectively, whereas endo-PGs lack these tails (Choi et al., 2004). As a result of their unique structures, exo-PGs can only remove galacturonic acid residues from the non-reducing ends of HG chains; endo-PGs catalyze the random hydrolytic cleavage of α-1,4 glycosidic bonds in HG chains; and rhamno-PGs catalyze the hydrolytic cleavage of α-1,2 glycosidic bonds randomly within or from the non-reducing ends of rhamnogalacturonan-I main chains (**Figure 1A**) (Markovič and Janeček, 2001; Park et al., 2010).

### Classification and Molecular Evolution of Polygalacturonases

As mentioned above, PGs can be divided into exo-PGs, endo-PGs and rhamno-PGs, based on their modes of hydrolysis. PGs of these different types emerged at different times during plant evolution. Rhamno-PGs, which are regarded as the earliest type, appear in both algae and land plants, and endo-PGs exist across land plants, whereas exo-PGs only appear in angiosperms (Park et al., 2010). The PG family in land plants had five common ancestral genes rather than a single one (McCarthy et al., 2014), which might be explained by this early divergence of rhamno-PGs and endo-PGs.

Using bioinformatics, PG genes can be grouped by their phylogenetic relationships. Two main classification systems have been proposed (**Figure 1B**) for analyzing these relationships according to amino acid sequence. The first system was put forward by Hadfield et al. (1998), who grouped three PG genes from melon (*Cucumis melo*) and 17 homologous genes from other plants and fungi into Clades A–C. In agreement with Hadfield’s system, Torki et al. (2000), Markovič and Janeček (2001), and Park et al. (2008) grouped PG genes into five clades: Clades A–E. Clades A, B, and C were found to have invariant conserved residues (Gly264 and Phe294 in Clade A, Asn104 in Clade B, and Lys176 in Clade C), revealing distinct structural characteristics in different clades. However, Clades D and E lack exclusively invariant residues (Markovič and Janeček, 2001). With the accumulation of genomic data for numerous species, PG classification entered into a new phase in which cluster analysis of all PG gene family members for a species could be performed. Park et al. (2010) divided 225 genes into six clades, which contain eight PG gene subfamilies from algae to angiosperms. Later, Liang et al. (2015) classified PG genes from five species ranging from algae to eudicots into seven clades, as supported by the classification of 557 PG genes from five grass and five eudicot species (Liang et al., 2016). Arabidopsis *QRT3* and its homologous genes in core angiosperms are grouped into the new clade (Clade G). The second classification system was proposed by Kim et al. (2006) and divided 125 PG genes from Arabidopsis and rice (*Oryza sativa*) into three classes, A–C. The subsequent studies that used this system also grouped 75 PG genes from poplar (*Populus trichocarpa*) and 100 PG genes from Chinese cabbage into three classes (Yang et al., 2013; Duan et al., 2016).

**FIGURE 2:**
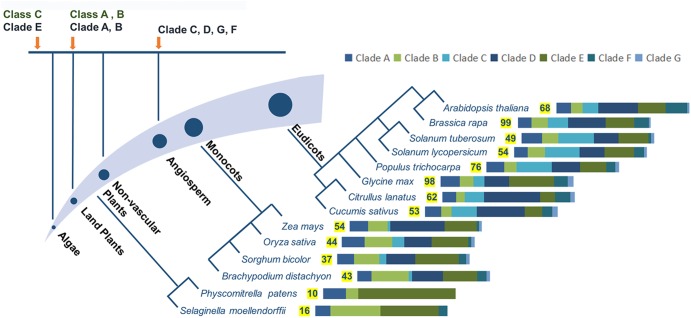
Evolutionary analysis of PG genes, numbers of family members, and proportions of different clades in representing species. Different clades or classes emerged at different periods during evolution, with appearance times indicated by orange arrows. PG family members are shown for different species of land plants, with significant changes in the proportions of different clades. Different bar lengths represent different proportions of clades. The numbers of family members in representative species are from reported studies (Tyler et al., 2010; McCarthy et al., 2014; Sénéchal et al., 2014; Yu et al., 2014; Liang et al., 2015, 2016) with some modifications.

Each classification system has advantages and shortcomings for analyzing molecular evolution. The first system is more suitable for analyzing the emergence of PG genes over time and the compositional changes in PG families. PG genes in different clades emerged at different times (**Figure 2**). Based on this system, PG genes in Clade E exist from algae to angiosperms, those in Clades A and B appear in land plants, and the genes in Clades C, D, F, and G only appear in flowering plants (Park et al., 2010). Clade emergence shows a pattern that is consistent with species evolution (**Figure 2**). The dominant clade in non-vascular plants is Clade E, which diversifies into Clades B, D, and E in monocots. The proportions of Clades B and E decrease in eudicots [except in soybean (*Glycine max*)], and Clades C, D and F are instead predominant. Further analysis shows that Clade D is the principal clade in Cruciferae, Cucurbitaceae, and Solanaceae, whereas the most-represented clades in poplar and soybean are Clades C and E, respectively. The second system also reveals different appearance times for different PG classes. However, PG genes within the same class also emerged at different times in this system. For example, the two major subgroups of Class A, A1, and A2, exist in angiosperms and non-vascular plants, respectively (Duan et al., 2016), and the proportion of Class C is lower than Classes A and B in species from moss (*Physcomitrella patens*) to vascular plants. The representation of Class A outpaces Class B in moss and vascular plants, but the converse is true in lycophytes (*Selaginella moellendorffii*). The various expansion rates of PG classes were likely driven by different selection pressures on those classes, accounting for their different proportionalities across taxa (Kim et al., 2006; Yang et al., 2013; Wang et al., 2016).

The first classification system has the advantage of analyzing large numbers of PG genes, which contributes to a comprehensive understanding of the phylogenetic relationships between genes across multiple species. Neighbor-joining and maximum parsimony algorithms were mainly used in this system, enabling concise and fast calculations to analyze large quantities of sequence data. However, the increased amount of data might lead to lower confidence in the existence of a new clade and reduce statistical accuracy. Based on the first system, the number of PG family members shows an increasing trend from non-vascular plants to monocots to eudicots. Gene duplication and loss are both likely to be contributors to these differences in gene number. For example, more old and recent duplications exist in Clade B in grasses, likely explaining the larger numbers of genes in this family in grasses than in eudicots (Liang et al., 2016). The difference in wall composition between grasses and eudicots, wherein pectins are less abundant in grasses than in eudicots, is another likely explanation for the different scales of PG families (McCarthy et al., 2014; Liang et al., 2016). In comparison, the second system built high-value phylogenetic trees, mainly by applying maximum likelihood algorithms. Although 212 PG genes from five species were successfully classified in the second system (Yang et al., 2013), these time-consuming calculations are inadequate for analyzing complex phylogenetic relationships among PG genes across a large number of species.

Instead, the second system proposed by Kim has the potential to reveal orthologous relationships and ancestral PGs between two species. In total, 21, 39, and 54 common ancestral PG genes were found between Arabidopsis and rice, poplar, and Chinese cabbage, respectively (Kim et al., 2006; Yang et al., 2013; Duan et al., 2016). The difference in ancestral gene number can be explained by the phylogenetic distance between these species. Genes clustered in the same group are orthologs. Therefore, the possible biological functions of candidate PG genes from a given species can be inferred by referring to the function of orthologous genes from other species, if this information is available.

## Expression, Functions, and Mechanisms of Action of Polygalacturonases

### Expression Patterns of Polygalacturonase Genes

Numerous PG genes have been identified in various species. Here, we summarize PG genes with defined accession numbers and expression patterns as analyzed by qRT-PCR and/or promoter activity (**Table 1** and **Supplementary Table 1**). According to their expression patterns, these genes are divided into five groups: ubiquitously expressed in multiple organs, specifically expressed in flowers and pollen, expressed during fruit ripening, expressed at sites of organ abscission and dehiscence, and expressed in other organs. Expression patterns among different clades/classes and PG genes with close phylogenetic relationships are discussed below.

**Table 1:** Expression and functions of identified PG genes.

Gene	Accession number	Species	Expression	Gene function	PG activity	Reference(s)
*NIMNA*	At2g33160	*Arabidopsis thaliana*	Reproductive organ (flowers, pollen, and siliques)	Suspensor elongation	Not tested	Babu et al., 2013
*PGX1*	At3g26610	*Arabidopsis thaliana*	Seedlings, roots, leaves, and flowers	Flower organ patterning, hypocotyl elongation, leaf expansion	√	Xiao et al., 2014
*PGX2*	At1g78400	*Arabidopsis thaliana*	Seedlings and in roots, stems, leaves, flowers, and siliques	Hypocotyl elongation, leaf expansion, stem lignification, mechanical stiffening, and lodging	√	Xiao et al., 2017
*PGX3*	At1g48100	*Arabidopsis thaliana*	Hypocotyls, roots, and at lateral root initiation sites, cotyledons, rosette leaves, flowers, siliques, seed funiculi, and stomata	Root and hypocotyl elongation, rosette expansion, the shape and the stomatal size of cotyledons, the stomatal dynamic in true leaves	√	Rui et al., 2017
*QRT3*	At4g20050	*Arabidopsis thaliana*	Tapeta in tetrad stage	Degrading pollen mother cell wall to allow microspore separation	√	Rhee et al., 2003
*BcMF2*	ABW24665	*Brassica campestris*	Tapeta and pollen after tetrad stage	Pollen wall intine layer development	Not tested	Huang et al., 2009a
*LLP-PG*	AY834213	*Lilium longiflorum*	The late stage of pollen maturation	Degrading the walls of stylar cells to allow penetration of the pollen tube	√	Chiang et al., 2006
*BcMF6*	ACP74159.1	*Brassica campestris*	Flower buds from stage III to stage V, open flowers and green silique	Pollen maturation and pollen tube growth	Not tested	Zhang et al., 2008
*GmPG031*	AP011817	*Glycine max*	Flowers, pollen grains, and pollen tube	Flowers and silique development	Not tested	Wang et al., 2016
*MdPG1*	L27743	*Malus domestica*	Ripening fruit from ripening early stage	Fruit ripening, reduce cellular adhesion and influence fruit texture characters; leaf morphology, stomatal structure and function, and leaf attachment	√	Atkinson et al., 2002, 2012; Wakasa et al., 2006; Costa et al., 2010; Li et al., 2010
*FaPG1*	DQ458990	*Fragaria ananassa*	Ripening fruit of firmer cultivar	Fruit ripening	√	Villarreal et al., 2008, 2009; Pose et al., 2013
*PG*	Ca10g18920	*Capsicum annuum*	Softening fruit	Fruit softening	Not tested	Kim S. et al., 2014
*PGAZAT (ADPG2)*	AT2G41850	*Arabidopsis thaliana*	Abscission zone of the sepals, petals, and stamens of flowers; anther during microspore separation; dehiscence zone of maturing siliques	Flower tissues abscission; anther and silique dehiscence; pollen separation	√	Ogawa et al., 2009; González-Carranza et al., 2012
*ADPG1*	AT3G57510	*Arabidopsis thaliana*	Dehiscence zone of silique; seed abscission zones; anther before anthesis	Anther and silique dehiscence; pollen separation	√	Ogawa et al., 2009
*QRT2*	At3g07970	*Arabidopsis thaliana*	Abscission zones of sepal, petal, and stamen in flowers	Flower organs abscission; anther dehiscence; pollen separation	√	Ogawa et al., 2009
*PS-2*	EU111748	*Solanum lycopersicum*	Dehiscence zone of anther	Anther dehiscence	Not tested	Gorguet et al., 2009

#### Polygalacturonase Genes Among Different Clades Are Conserved and Diversified in Expression

Previous studies revealed that the expression patterns of PG genes are conserved in the same clade, but diversified between clades. PG genes in Clades A and B are expressed in fruits and abscission and dehiscence zones, and genes in Clade C are expressed in flowers (Hadfield et al., 1998). This pattern is supported by the expression of most identified genes. PG genes such as *PS-2*, *ADPG1*, *ADPG2*, *RDPG1*, and *SDPG* (**Table 1** and **Supplementary Table 1D**) are expressed during organ abscission and dehiscence, and belong to Clade B with the conserved motif FGAKGDG (Rodriguez-Llorente et al., 2004); PG genes such as *PcPG1*, *VvPG1*, *VvPG2* and *MAPG3*, which are expressed during fruit ripening, belong to Clade B, and *sPG* belongs to Clade A (**Supplementary Table 1C**). PG genes such as *BcMF2, BcMF6, BcMF9*, *BcMF16*, *BcMF24*, and *PGA4* (**Table 1** and **Supplementary Table 1B**), are expressed in flowers and pollen and belong to Clade C. However, *FaPG1*, which belongs to Clade C and has a cysteine residue conserved in pollen-specific genes, is expressed in the middle-late stage of fruit ripening and is involved in flesh softening (Villarreal et al., 2008).

Genes from the same PG clade can also sometimes show distinct expression patterns. Hadfield’s hypothesis that orthologous genes are expressed similarly has proven to be partly incorrect. Expression patterns of PG genes are conserved in Clades D and E, with expression in inflorescences and ubiquitous expression in multiple organs, respectively (Yu et al., 2014; Liang et al., 2015, 2016). These results illustrate that these PG genes play crucial roles during multiple developmental processes. Members of Clades C and F, which are mainly expressed in reproductive organs in grasses and eudicots, are also sometimes expressed during root and seed development. A few eudicot PG genes of Clade C show expression in roots and root nodules. In some members of Clade F, expression can be detected in embryos and developing seeds as well. However, expression patterns are diversified in other clades. Members of Clades A, B, and G are either expressed specifically in different organs or widely across various organs. Some Clade G members, for example Arabidopsis *QRT3* and its homologs, are expressed either in one specific organ (such as seeds, stems, flowers, or siliques) or in multiple organs (Liang et al., 2016). Cucumber (*Cucumis sativus*) PG genes in Clade A are related to fruit development. However, these PG genes are expressed ubiquitously instead of showing fruit-specific expression (Yu et al., 2014).

Based on Kim’s system, Classes B and C have particular expression patterns in moss, lycophytes, rice, Arabidopsis, and poplar. However, members of Class A, which contains PG genes from lycophytes to angiosperms, are expressed with variable patterns (Kim et al., 2006; Yang et al., 2013). The requirement of angiosperm PG genes to function in the flower, which is the unique organ of angiosperms, might partly explain the diverse expression patterns of Class A genes. This diversity also likely relies on lower selective pressure compared with other classes (Yang et al., 2013). Specific expression patterns for genes within Class A, the largest class, can be found in its subgroups (Kim et al., 2006; Duan et al., 2016). To explore the relationship between these two systems of PG classification in a model species, we analyzed the Arabidopsis PG family using Kim’s system (Kim et al., 2006) and Liang’s system (Liang et al., 2015). Interestingly, almost all PG genes within the same subgroup of Kim’s system are grouped into a specific clade of Liang’s system, and different classes are composed of certain clade(s) (**Figure 1C**). This relationship can explain the specific expression of Classes B and C, which only contain PG genes from the deeply conserved Clade E (**Figure 2**). The complex composition of Class A accounts for its various expression patterns. Hence, the classification system of Hadfield has some advantages in connecting expression patterns to possible functions.

It is noteworthy that the conservation of expression patterns for PG genes is relative. PG genes expressed in the same general organs can still differ in specific expression patterns when they are studied in more detail. For example, *PcPG1* and *PcPG3* are both expressed in pear (*Pyrus communis*) fruit, but are expressed at different stages of fruit ripening and function separately in affecting fruit ripening and flesh texture, respectively (Sekine et al., 2006). Promoter activity analysis of *ADPG2* and its three closest homologs reveal that three of these genes are specifically expressed in different organs, and that another one had no detectable expression, even though *ADPG2* and its three closest homologs are all grouped into Clade B (González-Carranza et al., 2007). Expression specificity is also supported by the expression patterns of PG genes in other species. For example, 16 fruit-specific PG genes in cucumber can be divided into three subgroups, with highest expression occurring for each subgroup at 3 days after pollination, 6–9 days after pollination, or 27 days after pollination, respectively (Yu et al., 2014). In Arabidopsis, 47 abscission-related PG genes have nine significantly different expression patterns during five stages of floral organ abscission (Kim and Patterson, 2006). The diversity of expression patterns in the same organ implies that these genes play different roles in the development of a single organ, but this idea requires further research to be validated.

#### Duplicated Polygalacturonase Genes Show Divergent Expression

Duplicated PG genes, which are highly similar in sequence, are more likely to have different expression patterns. These gene pairs result from duplication events such as whole genome duplication (WGD) and tandem duplication (TD) during plant evolution. Two tandemly duplicated PG genes, *PpendoPGM* and *PpendoPGF*, promote the softening of peach flesh, but only the latter is involved in stone adhesion (Gu et al., 2016). *MaPG1* and *MaPG2*, of which the cDNA sequences are 98% similar, are expressed in different organs: the former is expressed in roots, stem, leaves, and flowers, whereas the latter is expressed in the late stage of fruit ripening (Mbéguié-A-Mbéguié et al., 2009). PG paralog pairs generated by TD are more likely to be expressed differently than pairs generated by WGD. For example, based on microarray and RNA-seq-based data, paralogous PG pairs with different expression patterns constitute 71.4%, 75%, and 75% of pairs resulting from TD in rice, poplar, and cucumber (Kim et al., 2006; Yang et al., 2013; Yu et al., 2014), whereas PG copies with diversified expression patterns cover 56% and 67.8% of PG copies caused by WGD in poplar and soybean, respectively (Yang et al., 2013; Wang et al., 2016). Expression diversity of duplicated genes is also demonstrated by variable expression between paralogs in Arabidopsis and Chinese cabbage, which underwent WGD twice and tree times, respectively (Liang et al., 2015). The difference in expression of duplicated PG copies highlights the diversity of their functions and exemplifies the duplicate retention mechanisms known as neofunctionalization and subfunctionalization (Innan and Kondrashov, 2010).

### Functions of Polygalacturonases in Pectin Degradation, Cell Dynamics, and Plant Development

#### Polygalacturonases and Pectin Degradation

Homogalacturonan, which is the most abundant pectin domain, has been extensively studied in terms of its synthesis, modification, and degradation. HG is synthesized in the Golgi apparatus, and its polysaccharide chain is extended by galacturonic acid residues being added to the non-reducing end. Under the action of pectin methyltransferases (PMTs) and acetyltransferases (PATs), HG can be modified with methyl-ester or acetyl groups. Newly synthesized HG polymers are apportioned into vesicles and transported to the plasma membrane, and finally are secreted into the apoplast for incorporation into the cell wall (Ridley et al., 2001; Sénéchal et al., 2014).

In the wall, HG can be metabolized by the action of HG modifying-enzymes (HGMEs), and participates in the regulation of plant development and responses to external stimuli. PGs, pectin methylesterases (PMEs), pectin acetylesterases (PAEs), and pectin/pectate lyase-like proteins (PLLs, including pectin lyases and pectate lyases) all are HGMEs. Their coding genes have distinct expression patterns (Sénéchal et al., 2014), and they modify pectin in different ways. PMEs and PAEs remove modifications from the HG backbone, whereas PGs and PLLs cleave or depolymerize HG. More specifically, PMEs control the degree of methylesterification of HG by removing methyl-ester groups, resulting in negatively charged galacturonic acid residues (Jolie et al., 2010). PAEs hydrolyze *O*-acetyl groups and produce linear HG (Gou et al., 2012). PGs hydrolyze the α-1,4 glycosidic bonds of demethylesterified HG, releasing oligogalacturonides (OGs) or galacturonic acid monomers (Markovič and Janeček, 2001). In contrast to PGs, pectin lyases and pectate lyases can cleave the α-1,4 glycosidic bonds of methylesterified and demethylesterified HG, respectively, by β-elimination (Mayans et al., 1997; Herron et al., 2000).

**FIGURE 3:**
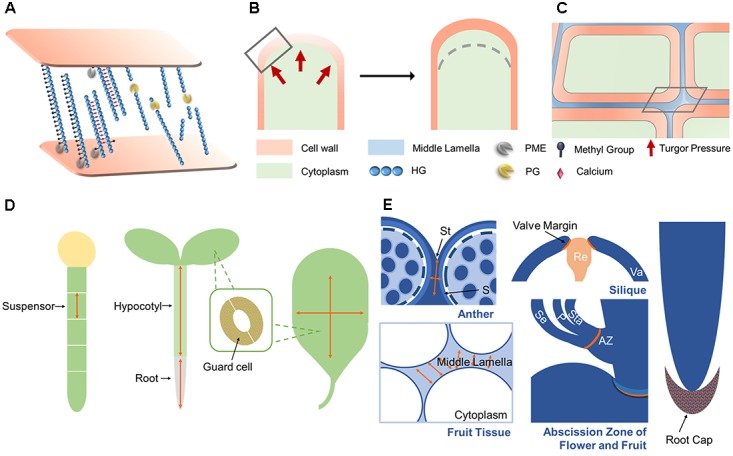
PG functions and mechanisms of action. **(A)** PG genes are involved in suspensor, root, and hypocotyl elongation, leaf expansion, and stomatal dynamics by functioning in cell expansion. **(B)** PG genes function in root call detachment, anther and silique dehiscence, organ abscission, and fruit softening by functioning in cell separation. **(C)** During cell expansion, the cell wall loosens due to the hydrolysis of pectins by PGs. Under intracellular turgor pressure, the cell wall expands outward and then cell elongated. **(D)** During cell separation, PGs function in the degradation of pectins within the middle lamella. As a result, adjacent cells are reduced in adhesion, then finally separate from each other. **(E)** The subcellular actions in **(C,D)** marked by gray rectangle are shown here. In the cell wall, de-esterified HG produced by PMEs can be crosslinked by calcium, stiffening the wall, or cleaved by PGs, resulting in wall loosening. In the middle lamella, PGs hydrolyze de-esterified HG in the same way as in the cell wall. Activity sites are noted in orange and the expansion direction is indicated by orange arrows in **(A,B)**. A lighter shade of cell wall and middle lamella means looser wall integrity in **(C–E)**. St, stomium; S, septum; Va, valve; Re, replum; Se, sepal; P, petal; Sta, stamen; AZ, abscission zone.

Models of HG remodeling have been proposed that take distinct cell wall microenvironments into consideration (Sénéchal et al., 2014; Hocq et al., 2017). In an acidic cell wall context, HG that is randomly demethylesterified by acidic PMEs can be a substrate for PGs. Basic PMEs, which can be easily trapped by free carboxyl groups in this circumstance, would inefficiently remove methyl-ester groups from HG. In a slightly alkaline context, H^+^ diffuse into the cytoplasm by producing IAAH, due to in the absence of auxin (IAA^-^). As a result, the cell wall can be high in the concentrations of ionic cations. Under this condition, basic PMEs play the main role in HG de-methylesterification, whereas acidic PMEs will be charged and unable to bind HG. Continuous stretches of demethylesterified HG can crosslink via Ca^2+^ to form stable “egg-box” structures, which restrict cell wall loosening (**Figure 3**). Other regions of HG can be hydrolyzed by PGs, releasing OGs. HG has been proposed to go through a cycle of crosslinking, modification, cell wall loosening, and cell growth (Sénéchal et al., 2014; Boyer, 2016; Hocq et al., 2017).

#### Polygalacturonase Mechanisms of Action in Cell Dynamics

Cell proliferation, expansion, and separation form the foundation of plant morphogenesis and development. During cell expansion and separation, pectin is degraded in the primary cell wall and middle lamella, decreasing cell wall stiffness and increasing wall fluidity. The identified roles of PGs in plant development reveal that they can function in both cell separation and expansion. Here, we summarize the possible mechanisms of action of PGs in cell dynamics.

Separation between cells in the abscission zone (AZ) results in organ abscission and dehiscence. After receiving the abscission signal in the AZ, the cell wall and middle lamella begin to detach via the action of cell wall degrading enzymes (**Figures 3A,C**). Cells separate along the middle lamella, allowing for organ separation and dehiscence (Kim et al., 2015). After organ detachment, cells in AZ can elongate, implying that cell elongation is also involved in organ abscission (Patterson and Bleecker, 2004). AZ-expressed PG genes function in root cap detachment, anther and fruit dehiscence, leaf shedding, and fruit maturation, highlighting their central roles in cell separation (**Figure 3E**). Crosslinks between adjacent cells are formed under the control of Ca^2+^, and cell adhesion is maintained by HG methylesterification (Daher and Braybrook, 2015). During cell separation, PMEs remove the methyl-ester groups from HG to provide substrates for PGs. PGs can then cleave HG backbones and reduce the HG-mediated cell adhesion that is maintained by Ca^2+^. Finally, cells detach from each other (**Figures 3A,C**) (Daher and Braybrook, 2015). This model is supported by the possible role of MdPG1 during leaf shedding. In MdPG1 overexpression lines, more abundant low-esterified pectins are found in AZs with increased PG activity in leaves (Atkinson et al., 2002). Noticeably, tetrad separation is a special case of tissue separation. Instead of cell separation, the release of microspores results from the degradation of the primary pollen mother wall. PGs like QRT3, which are secreted from tapetum cells during early microspore stage, are responsible for micropore separation by degrading this cell wall (Francis et al., 2006).

Cell expansion is required by numerous developmental processes such as suspensor elongation, hypocotyl elongation, stomatal opening and closure, and leaf expansion (**Figure 3D**). Therefore, PG genes like *POLYGALACTURONASE INVOLVED IN EXPANSION* (*PGX*) genes, which affect the elongation and expansion of several organs, might have their mechanism of action explained as facilitating cell expansion (Xiao et al., 2014, 2017; Rui et al., 2017). Lower demethylesterified HG level, higher PG activity and smaller HG size in *PGX3* overexpression lines provide further evidence for the functions of PGs in regulating stomata dynamics and leaf expansion (Rui et al., 2017). The aforementioned fact that cells in the AZ elongate after organ shedding (Patterson and Bleecker, 2004) suggests that abscission-related PGs might influence both cell separation and cell expansion. Cell expansion can be regulated by intracellular turgor pressure and mechanical interactions between cell wall components. The direction of cell expansion is influenced by both the orientation of cellulose microfibrils (Cosgrove, 2005; Palin and Geitmann, 2012) and the modification of pectins (Peaucelle et al., 2015). Turgor pressure, in combination with increased cell wall extensibility, is thought to be the driving force for cell expansion (**Figure 3B**). Pectins affect cell expansion by modulating the mechanical properties of cell walls (Peaucelle et al., 2011; Kozioł et al., 2017). By effecting pectin degradation, PGs are hypothesized to cause a decrease in cell wall stiffness (**Figures 3A,B**), which is supported by the identified role of *PGX2* in regulating the mechanical properties of stems (Xiao et al., 2017). PGs might also function in cell expansion by modulating the directionality of expansion. A mutant Arabidopsis line resistant to the drug cobtorin can suppress the cell-swelling phenotype induced by cobtorin, due to the overexpression of a PG gene in this line. Exogenously supplied PGs can also rescue this phenotype in Arabidopsis, restoring the deposition of cellulose microfibrils in parallel with cortical microtubules with the help of PME (Yoneda et al., 2010). Exactly how PGs might influence expansion direction requires further study.

It is noteworthy that PGs might participate in cell and tissue patterning. PME5, which works upstream of PGs, plays a key role in controlling phyllotactic patterning (Peaucelle et al., 2008). Changes in the degree of methylesterification of pectins can enhance cell wall loosening, which underlies organ initiation (Peaucelle et al., 2011). One PME inhibitor protein, PMEI3, can suppress morphogenesis in inflorescence meristems (Peaucelle et al., 2008). The deformed pollen grains that result from abnormal intine formation in *BcMF2*, *BcMF9* antisense lines indicate that PGs might also function in determining cell morphology during pollen development. However, whether PGs act in organ patterning through regulating cell wall mechanics directly or by changing wall integrity signaling remains unknown.

#### Biological Functions of Polygalacturonase Genes in Plant Development and Fruit Ripening

The functions of PG genes, which have been identified by mutants or transgenic lines (**Table 1**) or have in some cases been inferred from their expression patterns (**Supplementary Table 1**), reveal their significant roles in both vegetative and reproductive development in plants. Ubiquitously expressed PG genes have been identified as functioning in multiple processes during development, which might be important for plant survival. For instance, the expression of Arabidopsis *POLYGALACTURONASE INVOLVED IN EXPANSION1* (*PGX1*) is detected in seedlings, roots, leaves, and flowers (Xiao et al., 2014). Overexpression of this *PG* leads to significant elongation of etiolated hypocotyls and enhanced rosette leaf expansion. Its functions in cell and tissue expansion are supported by the opposite phenotypes in a *pgx1* knockout mutant. In flower development, abnormal angles between flower primordia and extra petals are found in both overexpression lines and mutants, which indicates that this *PG* might function in floral organ patterning (Xiao et al., 2014). Overexpressing Arabidopsis *POLYGALACTURONASE INVOLVED IN EXPANSION2* (*PGX2*) leads to faster flowering and enhanced stem lignification with enhanced tensile stiffness but lower bending stiffness, as well as enhanced etiolated hypocotyl length and rosette leaf area (Xiao et al., 2017). *PGX2* represents the first *PG* gene that has been found to regulate stem lignification. Arabidopsis *POLYGALACTURONASE INVOLVED IN EXPANSION3* (*PGX3*) also functions in rosette expansion and root elongation (Rui et al., 2017). Overexpressing this PG gene also causes larger stomatal pores in cotyledons, whereas its knockout mutant has the opposite phenotype. Without influencing stomatal dimensions in true leaves, *PGX3* asymmetrically affects mature stomatal opening and closure (Rui et al., 2017). Interestingly, despite their common functions in rosette leaf expansion, *PGX1*, *PGX2*, and *PGX3* are not closely related in the phylogeny of Arabidopsis PGs (McCarthy et al., 2014).

How PG genes influence pollen development and fertilization has been a recent research priority. The expression of PG genes in tapeta, pollen grains, stigmas and pollinated pistils implies their role in tapetum degradation, pollen maturation, pollen tube growth, and pollination, as evinced by their functional characterization (**Table 1**). Suppressing the expression of *QRT3* in Arabidopsis and *BcMF6* in Chinese cabbage interferes with microspore separation after the tetrad stage (Rhee et al., 2003; Zhang et al., 2008). In addition, the latter mutant has smaller floral organs and a lower pollen germination rate caused by the disruption of microspore maturation (Zhang et al., 2008). RNA antisense lines of Chinese cabbage *BcMF2* and *BcMF9* show disturbed development of the pollen wall intine layer and of the pollen tube wall that is the continuation of the intine layer (Huang et al., 2009a,b). The downregulation of *BcMF2* causes pollen deformity and balloon-like swelling in the pollen tube tip, along with premature tapetum formation (Huang et al., 2009a). Downregulating *BcMF9* influences pollen wall exine layer formation as well (Huang et al., 2009b). When a soybean PG is heterologously overexpressed in Arabidopsis, inflorescence mortality is over 50%, and siliques and seeds significantly decrease in number (Wang et al., 2016). *NIMNA*, which plays a role in early embryo cells and suspensor elongation, is the only Arabidopsis PG gene that has been identified as functioning in embryo development (Babu et al., 2013).

Early studies examined the relationship between PGs and fruit development and mainly focused on the activity of PG proteins and their responses to exogenous stimuli. In recent studies, fruit-related PG gene functions have gradually attracted more attention, especially in species with edible fruit. The suppression of *FaPG1* leads to significant increase in strawberry (*Fragaria ananassa*) fruit firmness (Pose et al., 2013). A similar change is also found in *PG1* suppression lines of apple (*Malus* × *domestica*), with an enhancement of cell adhesion (Atkinson et al., 2012). Despite *MdPG1* showing fruit-specific expression, its overexpression lines display various novel phenotypes in vegetative growth, such as the silvery colored leaves, earlier leaf shedding, and abnormal stomatal structure (Atkinson et al., 2002). Its function in organ abscission is also supported by heterologous expression studies in Arabidopsis. Overexpressing *MdPG1* in Arabidopsis can drive early silique dehiscence, whereas the siliques of *MdPG1* antisense lines do not open normally, possibly due to the downregulation of endogenous PGs (Li et al., 2013). In non-softening fruit species like pepper (*Capsicum annuum*), PG genes perform crucial functions as well. A point mutation in one pepper PG gene (CA10g18920) in wild type downregulates its expression by generating a premature stop code in its 3′ splicer accepted site. This results in lower water-soluble pectin levels compared with a *soft flesh* mutant, preventing aberrant fruit softening (Kim S. et al., 2014).

Abscission and dehiscence help to remove old, impaired and infected tissues as well as release leaves, floral organs, pollen or seeds, and are of great value in maintaining normal growth and reproduction. PG genes have been found to play a pivotal role in this process (**Supplementary Table 1D**). Mutation of tomato *PS-2* in intron recognition splice sites leads to non-dehiscent anthers and functional sterility (Gorguet et al., 2009). Similarly, an Arabidopsi*s adpg1 adpg2 qrt2* triple mutant is delayed in anther dehiscence (Ogawa et al., 2009). This phenotype is not found in double mutants or single mutants, indicating that anther dehiscence is co-regulated by these three PG genes. However, non-dehiscent siliques are found in single mutants of *adpg1*, *adpg2* and more prevalently in an *adpg1 adpg2* double mutant. *ADPG1* and *ADPG2* potentially function in silique dehiscence by hydrolyzing only a small amount of pectin (Ogawa et al., 2009).

Polygalacturonase genes also participate in biotic and abiotic stress responses in plants. These findings have been nicely summarized by Sénéchal et al. (2014). When treating strawberry fruit with resistance inducers like chitosan, benzothiadiazole or a mixture of calcium and organic acids, the expression of PG genes changes significantly (Landi et al., 2014). In the same study, when stimulated by abiotic stresses, the expression of PG genes is up-regulated, which might elicit responses to environmental stresses (Liu et al., 2014).

## Regulatory Networks of Polygalacturonase Genes and Encoded Proteins

### Regulation of Polygalacturonase Gene Expression

**FIGURE 4:**
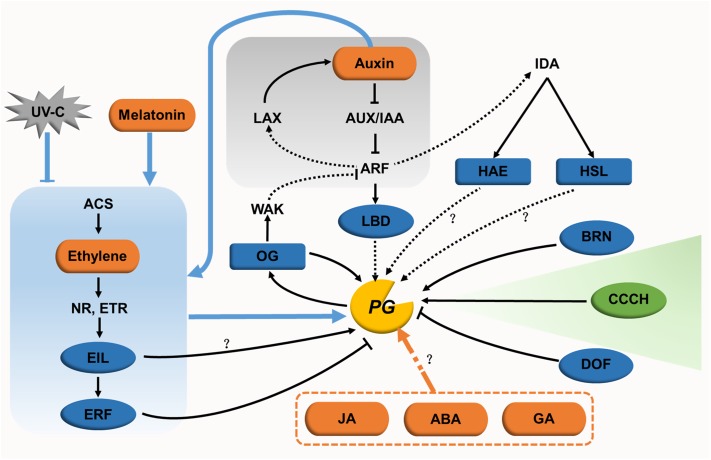
Regulatory networks for PGs in cell expansion and separation. In cell expansion, only one regulator (green oval) has been identified. The regulatory network for this cell dynamic processes remain unclear. In cell separation, hormones (orange ovals) and their signaling pathways (with gray or blue background), transcription factors (blue ovals), other endogenous factors (blue rectangles), and the exogenous factor UV-C (gray polygon) form a genetic regulatory network for PGs. The direct or indirect action of some regulators needs to be further studied (black dotted lines). The regulatory pathways of JA, ABA, and GA in relation to PGs also need to be characterized further.

The expression patterns of PG genes are regulated by various factors such as hormones, transcription factors, and environmental factors. These factors can function separately or be combined to form regulatory networks. Based on reported gene regulation in processes like lateral root initiation, root cap detachment, fruit ripening, and organ abscission, we summarize the regulatory network of PG genes in cell separation in **Figure 4**.

Lateral root initiation requires the primordium to emerge successively through the endodermal layer, cortical layer, and epidermal layer. PGs likely act to separate cells in the cortical and epidermal layers, contributing to the elongation of the primordium (Swarup et al., 2008; Kumpf et al., 2013). During this stage, a regulatory network (**Figure 4**) is formed centering on the auxin signal pathway and connecting with transcription factors, receptor-like kinases and OGs. Auxin influx carrier LIKE-AUXIN3 (LAX3) and its downstream auxin-inducing transcription factor LATERAL ORGAN BOUNDARIES DOMAIN18 (LBD18) indirectly increases the expression of *AtPG10* (Swarup et al., 2008; Kumpf et al., 2013; Lee et al., 2015). INFLORESCENCE DEFICIENT IN ABSCISSION (IDA) is the key regulator of cell separation, the expression of which is induced by AUXIN RESPONSE FACTOR7. After receiving a signal from IDA, the receptor-like kinase HAESA promotes the expression of *ADPG2* in lateral root development (Kumpf et al., 2013). However, whether HAESA regulates *ADPG2* directly has not been reported. OGs, the product of pectin degradation by PGs, might in turn control PG gene expression (Moscatiello et al., 2006). Exogenous OGs inhibit lateral root formation by suppressing the expression of auxin synthesis- and signaling-associated genes, such as *IAA5* and *SAUR16* (Savatin et al., 2011). Taking these results together, the interaction between auxin and PG-induced OGs is assumed to form a feedback regulation network (Ferrari et al., 2013). In this model, PG expression is induced by auxin, and the resulting PG proteins then release OGs in the apoplast. The accumulation of OGs in turn perturbs auxin responses. In root cap detachment, the transcription factor BEARSKIN1 directly binds to the *ROOT CAP POLYGALACTURONASE* (*RCPG*) promoter at a region containing a NAC-BINDING ELEMENT motif. The activation of *RCPG* accelerates cell detachment at the root cap (Kamiya et al., 2016).

In the process of fruit ripening, ethylene synthesis and signaling are crucial for regulating PG expression. Upon treatment with exogenous ethylene in the full bloom stage, expression of apple *PG1* is upregulated in the pericarp, leading to fruit softening (Tacken et al., 2010). The same expression pattern is found for the ethylene synthesis gene *ACC OXIDASE1*, which indicates that ethylene might regulate *PG1* expression. This idea was confirmed by the finding that EIN-LIKE3 transactivates the expression of *PG1* in transient assays (Tacken et al., 2010). Furthermore, ETHYLENE RESPONSE FACTOR9 (ERF9) in papaya (*Carica papaya*), which functions downstream of EIN3-LIKE, represses the expression of *CpPG5* in fruit ripening by specifically binding to its promoter at a GCC-BOX motif. Its repressive role relies on the ERF-ASSOCIATED AMPHIPHILIC REPRESSION motif within ERF9 (Fu et al., 2016). Auxin functions in this process in concert with ethylene. With naphthalene acetic acid (NAA) treatment, the effect of an ethylene repressor is enhanced in downregulating the expression of ethylene-independent *MdPG2* in the cortex and abscission layer, and ethylene-dependent *MdPG1* in the abscission layer as well. However, NAA conversely upregulates the expression of *MdPG1* in the cortex, which results in the increased accumulation of ethylene in this layer (Li and Yuan, 2008). Apart from auxin, UV-C and melatonin also interact with ethylene in regulating PG genes. Treating postharvest tomato fruits with UV-C decreases the synthesis of ethylene, and suppresses the expression of PG genes together with other cell wall modifying genes during fruit softening (Bu et al., 2013). These data reveal that the regulatory mode of UV-C may be through the action of ethylene. Melatonin influences ethylene signaling in postharvest tomato ripening by promoting the expression of ethylene related genes (such as *NR*, *ETR4*, *EILs*, and *ERF2*), causing the upregulation of PG genes (Sun et al., 2015). ABA and GA also take part in this regulatory network. ABA treated tomato is downregulated in the expression of *SlPG* and other genes encoding wall-modifying enzymes, showing a delay in fruit softening (Sun et al., 2012). However, strawberry fruit treated with ABA showed slightly increased *FaPG1* expression without influencing its protein activity (Villarreal et al., 2009). GA affects flower blooming and fruit maturity in grapes (*Vitis labrusca* × *V. vinifera*) with two 230-fold upregulated PG genes (Cheng et al., 2015). However, the regulatory pathways for these two hormones need to be studied further.

During organ abscission, jasmonic acid (JA), transcription factors, and receptor-like kinases are involved in regulating PG expression. Microarray transcriptome data of a JA mutant treated with JA show that the expression of *ADPG1*, *ADPG2*, and *QRT2* are upregulated 10-fold upon JA treatment (Mandaokar et al., 2006). GUS activity of the *QRT2* promoter is detected in the anther of a JA mutant treated with methyl jasmonate (Ogawa et al., 2009). Taken together with the delayed dehiscence of anthers and siliques in the *adpg1 adpg2 qrt2* mutant, the non-dehiscent anther of the JA mutant may be caused by decreased PG gene expression (Ogawa et al., 2009). Additionally, the transcription of *ADPG2* in the AZ might be regulated directly via the binding of the transcription factor FOR DNA BINDING WITH ONE FINGER4.7 to the AAAG *cis*-regulatory element of its promoter (Wei et al., 2010). During lateral root emergence, both HAESA and HAESA-LIKE2 act downstream of IDA. These two kinases promote the expression of *ADPG2* in floral organ AZ via regulatory mechanisms that are currently unclear (González-Carranza et al., 2012).

Surprisingly little is known about the regulation of PG expression in cell expansion. Only a plant-specific tandem CCCH zinc-finger C3H14 in Arabidopsis has been demonstrated to affect cell elongation by binding to the AU-RICH ELEMENT motif of the *ADPG1* promoter and activating its transcription (Kim W.C. et al., 2014). The roles of hormones, other transcription factors and environmental factors in regulating PG-mediated cell expansion need to be studied further.

### Regulation of Polygalacturonase Biochemical Activities

External factors, other proteins, and the apoplastic microenvironment can all act as regulators of PG biochemical activities. PG activities can be controlled by temperature, pressure, exogenous polyamine and expansin (Crelier et al., 2001; Fachin et al., 2002; Martinez-Tellez et al., 2002; Karakurt and Huber, 2003; Yuan et al., 2011; Nardi et al., 2013). UV-C, which was mentioned above as a transcriptional inhibitor, delays tomato fruit softening by negatively affecting PG activities (Barka et al., 2000). Hormones can also influence PG activities. During strawberry fruit ripening, exogenous NAA and an inhibitor of ethylene perception (1-MCP) decrease the expression of *FaPG1* and the activity of FaPG1, resulting in the delay of ripening. However, this phenotype is not necessarily caused by the difference in gene expression, because treatment with GA_3_ reduces the activity of FaPG1 without significantly changing gene expression (Villarreal et al., 2009).

The wall microenvironment influences PG activities mainly through cations and pH. Ca^2+^ deficiency in potato leaves leads to a dramatic increase in PG enzyme activity (Seling et al., 2000), and the inhibitory role of Ca^2+^ for PG activities was also supported by treating grape berries with 1 mM Ca^2+^ (Cabanne and Doneche, 2001). In mango (*Mangifera indica*) fruit, cations like Li^+^, Mg^2+^, and a high substrate (polygalacturonic acid) concentration suppress PG activities, whereas Fe^3+^ activates PG activities (Prasanna et al., 2006). PGs show high activity within physiological pH ranges. Two PG isoforms isolated from avocado (*Persea americana*) fruit mesocarp show highest activity when the pH is 6.0 (Wakabayashi and Huber, 2001). The influence of pH on PG activity can be altered by intercellular cations. The optimal pH for PG activity in tomato is 4–4.5 with NaCl, and changes to 5–6 with KCl (Chun and Huber, 1998). pH and Ca^2+^ are also likely to play complex roles in pectin degradation by influencing the action of PMEs and the formation of PG substrates.

Posttranslational modifications and interacting proteins can also influence PG activities. Polygalacturonase inhibiting proteins (PGIPs) can specifically bind and inhibit PG activities. Studies on the PGIP–PG interaction have been well summarized before (Kalunke et al., 2015). The β subunit of a tomato fruit polygalacturonase inhibits the interaction between PG and pectins (Zheng et al., 1992). To figure out whether a posttranslational modification works in affecting PG activities, phenylalanine residues within the FxxY sequence in a β subunit were modified to α, β-didehydro-Phe (Sergeant et al., 2017). This modification promoted βPG binding to pectins. It also contributed to the interaction of βPG with the catalytic subunit of PG (Sergeant et al., 2017). In addition, an 8-kD non-specific lipid transfer protein, ACTIVATOR, which is contained in a PG1 multiprotein complex, might change the form of associated PG into a heat-stable and active one (Tomassen et al., 2007). N-terminal prosequences have been hypothesized to control PG activity through influencing protein folding or protein inactivation (Hadfield and Bennett, 1998; Torki et al., 2000), but in at least some cases, the N-terminal prosequence instead controls the secretion of PG to the cell wall, as demonstrated in *Pichia pastoris* and Arabidopsis (Dal et al., 2001; Babu et al., 2013). To explore the posttranslational modifications of PG activity, an advanced approach for producing custom-folded proteins is required for further studies.

## Conclusion

Major advances in our knowledge of the expression patterns, biological functions, and mechanisms of action of PGs have deepened our understanding of their roles in vegetative growth, pollen development, fruit ripening, and organ abscission. Although PGs are not the only pectin hydrolases, these studies provide convincing evidence for their unique importance in plant development. Based on genome data and current classification methods, we have a much clearer knowledge of PG structural characteristics, evolution, and expression patterns. However, shortcomings exist for each classification system, which calls for a better system that combines convenient calculations of phylogenetic relationships with high confidence values when dealing with PG families at large scales. PG genes with diverse expression patterns in the same tissues need to have their biological functions pinpointed for a better understanding of their individual activities and retention mechanisms. To illuminate the ways in which PGs function in developmental processes, a comprehensive regulatory network needs to be built based on well-studied pathways that regulate PG expression and activity. As a wider suite of dyes and antibodies become available for observing pectin dynamics (Mravec et al., 2014, 2017; Pattathil et al., 2015; Anderson, 2016; Rydahl et al., 2018; Voiniciuc et al., 2018), we can dive into the mechanisms of PG action in cell wall dynamics. These and other studies can help us gain insights into the role of pectins in plant cell wall construction and dynamics as well as their relationships with other cell wall components.

## Author Contributions

YgY, YjY, and YL wrote the manuscript. CA and JC revised the paper. All the authors read and approved the final version of the manuscript.

## Conflict of Interest Statement

The authors declare that the research was conducted in the absence of any commercial or financial relationships that could be construed as a potential conflict of interest.
