# Supplementary material for: A Profusion of Molecular Scissors for Pectins: Classification, Expression, and Functions of Plant Polygalacturonases
Source: Front Plant Sci. 2018 Aug 14;9:1208. doi: 10.3389/fpls.2018.01208 (PMC6102391; doi:10.3389/fpls.2018.01208)
Supplement: Supplementary file 1 [file Table_1.DOCX]

Supplementary Material

**Last but not least: Classification, expression, and functions of plant polygalacturonases**

**Yang Yang^1,2^, Youjian Yu^1,3^, Ying Liang^1,2^, Charles T. Anderson^4, 5^, Jiashu Cao^1,2*^**

*** Correspondence:** Prof. Jiashu Cao**:** jshcao@zju.edu.cn

1. **Supplementary Table**

**Table 1. Expression and possible functions of identified PG genes.**

（A）PG genes with ubiquitous expression patterns and their functions.

| **Gene(s)** | **Accession Number** | **Species** | **Expression** | **Gene function** | **Reference(s)** |
| --- | --- | --- | --- | --- | --- |
| *CkPGA* | AF152753.1 | *Actinidia chinensis* | Fruit, flower buds, petals, senescent petal | Flower development | Wang *et al*., 2000 |
| *CkPGB* | AF152755.1 | *Actinidia chinensis* | Fruit, flower buds, petals, senescent petal | Flower development | Wang *et al*., 2000 |
| *MAPG1* | AF311881 | *Musa accuminata* | Roots, stems, leaves and flowers | Undefined | Asif and Nath, 2005 |
| *GmPG11* | DQ340401 | *Glycine max* | *Glycine max* cyst nematodes-colonized roots, flowers, and ethylene induced abscission zones | Undefined | Tucker *et al*.,2007 |
| *PdPG1* | DQ375247 | *Prunus domestica* | Flower and fruit development | Fruit ripening and seed mature | Iglesias-Fernandez *et al*., 2007 |
| *BnQRT3* | EE392283 | *Brassica napus* | Floral organs, leaves, stem and pollen | Pollen development and flora organ abscission | Wan *et al*., 2010 |

（B）PG genes with specific expression in flower and pollen development and their functions.

| **Gene(s)** | **Accession Number** | **Species** | **Expression** | **Gene function** | **Reference(s)** |
| --- | --- | --- | --- | --- | --- |
| *pSgPG1* | AB029457 | *Salix gilgiana* | Mature pollen grains after microspore  mitosis | Pollen development | Futamura *et al*., 2000 |
| *pSgPG4* | AB029460 | *Salix gilgiana* | Mature pollen grains after microspore  mitosis | Pollen development | Futamura *et al*., 2000 |
| *TAPG4* | AF001002 | *Solanum lycopersicum* | The upper third of mature pistils | Pollination | Hong and Tucker, 2000 |
| *TPG7* | AF072732 | *Solanum lycopersicum* | The upper third of pistils from opened  and unopened flowers | Pistil development or pollination | Hong and Tucker, 2000 |
| *PGA4* | NM100158 | *Arabidopsis thaliana* | Tapeta at the bicellular pollen stage  and in tricellular pollen | Pollen development | Ariizumi *et al*., 2002 |
| *MsPG11* | AY219848 | *Medicago truncatula* | Mature and germinating pollen grains | Pollen tube elongation | Rodriguez-Llorente *et al*., 2004 |
| *HvPG1* | X57627 | *Hordeum vulgare* | Gametogenesis after microspore division | Pollen development | Pulido *et al*., 2009 |
| *BcMF9* | ABN13878.1 | *Brassica campestris* | Tapeta and microspores during  the late stages of pollen development | Pollen wall intine layer and exine layer formation | Huang *et al*., 2009b |
| *OsPGT1* | AP014959 | *Oryza sativa* | Anther tissue at the booting stage | Anther development | Kato *et al*., 2010 |
| *BcMF17* | DY654330 | *Brassica campestris* | Male floral tissues from the anther  primordia stage to the mature pollen stage | Pollen wall formation | Zhang *et al*., 2011 |
| *BcMF16* | HM156046 | *Brassica campestris* | Tapeta and pollen at the tetrad stage | Pollen wall formation | Zhang *et al*., 2012 |
| *CpPG1* | HQ232488 | *Cucurbita pepo* | Pollen and the rudimentary anthers  of bisexual flowers | Pollen development | Carvajal *et al*., 2014 |
| *BcMF24* | KF670977 | *Brassica campestris* | Microspores, tapeta at binucleate stage and mature pollen grains | Pollen development | Yu *et al*., 2014b |
| *BoMF25* | XM_013741365.1 | *Brassica oleracea* | Mature pollen grains | Pollen wall development | Lyu *et al*., 2015 |

（C）PG genes with specific expression in fruit ripening and their functions.

| **Gene(s)** | **Accession Number** | **Species** | **Expression** | **Gene function** | **Reference(s)** |
| --- | --- | --- | --- | --- | --- |
| *sPG* | AY282613 | *Fragaria ananassa* | Fruit ripening stage W1 and W2 | Fruit abscission | Redondo-Nevado *et al*., 2001; Salentijn *et al*., 2003 |
| *MAPG2* | AF311882 | *Musa accuminata* | Later stages of fruit ripening | Fruit senescing | Asif and Nath, 2005;  Mbéguié-A-Mbéguié *et al*., 2009 |
| *MAPG3* | AY603339 | *Musa accuminata* | Softening fruit | Fruit ripening and softening | Asif and Nath, 2005;  Mbéguié-A-Mbéguié *et al*., 2009 |
| *MAPG4* | AY603341 | *Musa accuminata* | Softening fruit | Fruit ripening and softening | Asif and Nath, 2005;  Mbéguié-A-Mbéguié *et al*., 2009 |
| *PcPG1* | *AB066350* | *Pyrus communis* | Ripening fruit | Fruit softening | Sekine *et al*., 2006 |
| *PcPG3* | AB067642 | *Pyrus communis* | Ripening fruit | Development of the melting texture | Sekine *et al*., 2006 |
| *OsPG* | DQ437502 | *Opuntia* sp. | Ripening fruit from ripening early stage (sensitive to ethylene, cold storage and wounding) | Fruit ripening | Rosas-Cardenas *et al*., 2007 |
| *VvPG1* | AY043233 | *Vitis vinifera* | Developing grape skin | Skin softening | Deytieux-Belleau *et al*., 2008 |
| *VvPG2* | EU078975 | *Vitis vinifera* | Developing grape skin | Trigger fruit ripening process | Deytieux-Belleau *et al*., 2008 |
| *MdPG2* | AB210897 | *Malus* × *domestica* | Fruit abscission zones and fruit cortex | Young fruit abscission caused by NAA | Li and Yuan, 2008; Zhu *et al*., 2008; Li *et al*., 2010 |
| *cpPG* | FJ007644 | *Carica papaya* | Pulp | Pulp softening | Fabi *et al*., 2009 |
| *MzPG* | EU139437 | *Manilkara zapota* | Postharvest ripening fruit | Decrease in fruit and flesh firmness | Kunyamee *et al*., 2010 |
| *CaPG* | FJ596175 | *Capsicum annaum* | Pericarp from green, color change  and fully ripened stages | Dissolution of middle lamella and disruption of  the fully ripened cell wall during fruit ripening | Ahmed *et al.*, 2011 |
| *CitPG* | EF185420 | *Citrus sinensis* | Fruit peel and pulp | Favorable enhancement of the fruit mastication trait and dissolution of protopectin during cell enlargement | Liu *et al*., 2011 |
| *PaPG* | HQ540310 | *Prunus armeniaca* | Postharvest ripening fruit | Fruit ripening | Leida *et al*., 2011 |
| *cpPG1* | ACH82233 | *Carica papaya .* | Softening fruit | Fruit ripening | Fabi *et al*., 2014 |
| *PpendoPGF* | CM007654.1 | *Prunus persica* | Ripening fruits | Melting flesh and stone adhesion | Gu *et al*., 2016 |
| *PpendoPGM* | CM007654.1 | *Prunus persica* | Ripening stage but not in ripening fruit | Controlling melting flesh | Gu *et al*., 2016 |
| *PbPG1 / PpPG1* | KC855751;  JN048111 | *Pyrus communis* | Softening fruit | Fruit rapid softening | Song *et al*., 2016 |
| *PbPG2 / PpPG2* | KC855752; JN048113 | *Pyrus communis* | Softening fruit | Fruit rapid softening | Song *et al*., 2016 |

（D）PG genes with specific expression in organ abscission and dehiscence and their functions.

| **Gene(s)** | **Accession Number** | **Species** | **Expression** | **Gene function** | **Reference(s)** |
| --- | --- | --- | --- | --- | --- |
| *PGAZBRAN* | AJ250919 | *Brassica napus* | Abscission zone of leaf and flower during shedding | Leaf and flower abscission | González-Carranza *et al*., 2002 |
| *RDPG1* | X95800 | *Brassica napus* | Dehiscence zones of siliques and anthers, floral abscission zones and stylar tissues; Branch points between stems and pedicel; Apical meristem of seedlings, petals, cotyledons and roots | Breakdown middle lamella during silique opening | Sander *et al*., 2001 |
| *SDPG* | AF434714 | *Glycine max* | Pod dehiscence zone of mature *Glycine max* | Breakdown middle lamella prior to dehiscence | Christiansen *et al*., 2002 |
| *BrPG1* | AJ428543 | *Brassica rapa* | Silique valves at the beginning of its desiccation | Silique shattering | Rodriguez-Gacio *et al*., 2004 |
| *EgPG4* | JX233616 | *Elaeis guineensis* | The base of the oil palm fruit | Fruit shedding | Roongsattham *et al*., 2012 |

（E）PG genes with expression in other organs and their functions.

| **Gene(s)** | **Accession Number** | **Species** | **Expression** | **Gene function** | **Reference(s)** |
| --- | --- | --- | --- | --- | --- |
| *MsPG3* | AJ620946 | *Medicago truncatula* | Root hair | Tip growth of root hair | Rodriguez-Llorente *et al*., 2003 |
| *ZePG1* | AB047928 | *Zinnia elegans* | Tracheary elements | Degradation of pectic substances  before lignification | Nakashima *et al*., 2004 |
| *GmPGN* | AI973769 | *Glycine max* | Nodule tissue | Undefined | Jeong *et al*., 2006 |
| *AtPG10* | At5g14650 | *Arabidopsis thaliana* | Lateral root primordia | Lateral root growth | Swarup *et al*., 2008 |
